# Quality of Life in Mexican Older Adults: Factor Structure of the SF-36 Questionnaire

**DOI:** 10.3390/healthcare10020200

**Published:** 2022-01-20

**Authors:** Susana Ivonne Aguirre, Martha Ornelas, Humberto Blanco, Perla Jannet Jurado-García, Elia Verónica Benavides, Judith Margarita Rodríguez-Villalobos, Carolina Jiménez-Lira, José René Blanco

**Affiliations:** Faculty of Physical Culture Sciences, Autonomous University of Chihuahua, Chihuahua 31000, Mexico; siaguirre@uach.mx (S.I.A.); mornelas@uach.mx (M.O.); pjurado@uach.mx (P.J.J.-G.); ebenavides@uach.mx (E.V.B.); jurodrig@uach.mx (J.M.R.-V.); cajimenez@uach.mx (C.J.-L.)

**Keywords:** quality of life, health, factorial structure, construct validation, structural equations

## Abstract

The evaluation of quality of life may enable researchers to produce information that may improve health care and the quality of older people’s lives. This research has two main goals: the first is to assess the psychometric properties of the SF-36 Health Questionnaire (construct validity and internal consistency), and the second, to calculate the factorial invariance of the questionnaire in two random, independent samples (i.e., cross-validation). The total sample consisted of 970 elderly subjects from the cities of Chihuahua and Monterrey, Mexico, with an average age of 71.18 (SD = 7.69). The factor structure of the SF-36 was analyzed through confirmatory factor analysis (CFA). The analyses show an adequate four-factor structure. The four-factor structure (Physical Function, Body Pain, Physical Role and Psychological Health) shows adequate reliability and validity indices. In addition, the results from the CFA analyses for the subsamples provide strong evidence of the stability of the four-factor structure. Future research should consider replicating the present findings in larger samples.

## 1. Introduction

The increase in life expectancy of the population, due to the decrease in fertility and increase in longevity, has produced progressive aging of the world population, leading to an aging society [1]. Aging is a process of gradual, irreversible, natural and progressive change that, over time, will bring alterations in physical and psychological abilities that will make it difficult for the individual to interact with their context [2].

Quality of life is an important component in determining health status; in the case of older adults, many physical aspects of quality of life, such as the energy to move, the absence of pain and the ability to carry out daily life activities, are particularly important; hence, the degree to which the dimensions of their quality of life are affected will vary depending on the environment, lifestyle and the degree of deterioration of their capacities [3,4].

The term “quality of life” can be analyzed from different perspectives, considering it as multicontextual [5] in such a way that quality of life can only be described and measured in individual terms of objective circumstances and subjective evaluations. The latter depend on the interpretations and evaluations that the individual makes about situations and conditions of the environment where they develop, and will depend on the attitude towards adverse situations and the satisfaction towards themselves and their life [6,7].

In turn, quality of life has been grouped into General Quality of Life and Health-Related Quality of Life (HRQL). The first is based on a definition that encompasses a feeling of wellbeing and happiness, without reference to health problems or disorders, while HRQL is part of a multidimensional approach with special attention to the functional effects of disease and its treatment on physical, mental and social functioning [8].

To improve the quality of life of older adults, understanding the links between biological aging, individual factors, and the dimensions of quality of life are important for the planning of health interventions and services [9].

Interest in measuring quality of life has grown substantially in recent years. Subsequently, different scales and questionnaires have been considered for their assessment. The SF-36 Health Questionnaire was found in the bibliographic review to be the most widely used questionnaire to assess health-related quality of life [10,11].

This instrument has been adapted for use in different contexts related to research studies, clinical and social interventions; it has been used in the general population and in populations with health problems [12,13,14,15,16], and has even been considered as a reference standard for the development or validation of other instruments that assess HRQL. In addition, the SF-36 has also been validated and translated in various countries and various languages [17,18,19,20].

In Mexico, some research has been conducted using the SF36 questionnaire to determine HRQL in specific groups of patients [21,22,23,24]. Likewise, there have also been some studies on the validation and adaptation of the SF-36 questionnaire, which has shown adequate psychometric properties [6,25,26]; however, these studies were not exclusively developed to assess the psychometric properties of the SF36 questionnaire in the elderly population.

In this context, the present instrumental study [27] has aimed to provide empirical support to the factor structure of the SF-36 Health Questionnaire (composed of items that assess both positive and negative health states) in the older Mexican population. This is justified by the importance of checking the factor structure of an instrument and its psychometric equivalence in different groups, since, in the context of intergroup comparison, it is essential to consider the need to carry out the adaptation of a psychological measurement instrument that meets all equivalence criteria, but above all to consider whether the same factor structure is applicable to different groups of subjects or, more generally, to different populations [28]. Therefore, this research has two main goals: the first is to assess the psychometric properties of the SF-36 Health Questionnaire (construct validity and internal consistency) and the second is to calculate the factorial invariance of the questionnaire in two random, independent samples (cross-validation).

## 2. Materials and Methods

### 2.1. Participants

A total of 970 participants were included in the sample: 701 women and 269 men, all elderly people from the cities of Chihuahua and Monterrey, Mexico. The participants were in the age group of 60 to 101 years with an average age of 71.18 and SD = 7.69. The sample was obtained through convenience sampling. The inclusion criteria for the current study were: being 60 years of age or older, residing in the cities in which the study was conducted, voluntarily agreeing to participate. People with symptoms of confusion or diagnosed with dementia or other serious psychiatric illness that prevented them from completing the questionnaire were excluded from the study. 

Two subsamples were created randomly from the total sample using IBM SPSS Statistics 21.0; this was done to carry out equivalent studies that allowed for cross-validation [29].

The first subsample was constituted by 482 participants: 339 women and 143 men. Participant age ranged from 60 to 94 years, (M = 71.11, SD = 7.44 years).

The second subsample included 488 participants: 362 women and 126 men. Participant age ranged from 60 to 101 years (M = 71.26, SD = 7.94 years).

### 2.2. Instruments and Variables

SF-36 Health Questionnaire adapted by Alonso, Prieto and Antó [30] consists of 36 items which detect positive and negative states centered on functional status and emotional wellbeing. The items are subdivided into eight dimensions: Physical Function (10 items); Vitality (4 items); Physical Role (4 items); Body Pain (2 items); General Health (5 items); Social Function (2 items); Emotional Role (3 items); and Mental Health (5 items). Additionally, the SF-36 includes a transition question on the change in overall health status from the previous year.

Participants responded on a Likert-type scale, where the number of response options varies from two to six points, depending on the item. For example, items 3 and 6 that measure physical function ranged from 1 (yes, I am very limited) to 3 (is not limited to anything); items which measure the physical role, such as 13 and 14, show dichotomous responses (1 is yes and 2 is no); items which measure corporeal pain, such as 22, present scores of 1 (nothing) to 5 (much); and finally, items such as 28 and 29 that measure mental health and vitality, respectively, present scores of 1 (always) to 6 (never). The items are inversely coded so that they have the same meaning.

In the present research, three modifications were made to the scale by Alonso, Prieto and Antó [30]:

First, the participant chooses among 11 possible responses on all items. The final version of the scale used in this research was combined with the original as described next: nothing (0), little (1, 2 and 3), regular (4, 5 and 6), much (7, 8 and 9) and very much (10). This first adaptation was carried out with the intention of obtaining a greater variability in the responses.

The second adaptation consisted of changing some words from items in the original version to provide more appropriate vocabulary for the Mexican cultural context.

The third adaptation included the participants completing the questionnaire using a computer (see Appendix A for an example of an item response screen). This modification was carried out to facilitate data storage without requiring prior coding, with greater precision and speed, thus, making the data collection faster and more precise than if paper and pencil were used.

### 2.3. Procedure

Elderly people were invited to participate in the study. Those who agreed to participate signed the informed consent form. The SF-36 was then completed using a computer (manager module of the instrument of the typical execution scales editor), in a single 45 min session. Before completing the instrument, participants were given a brief introduction regarding the relevance of the study; participants were also instructed on how to access the instrument.

Total honesty was required from participants and the confidentiality of the collected data was ensured. The first screens of the computerized version of the scale included instructions to complete the questionnaire (prior to the first item). Once the instrument was completed, participants were thanked for their collaboration.

The results were collected through the results generator module of the scale editor version 2.0 [31].

### 2.4. Data Analyses

As an initial stage in the analysis, means, standard deviations, skewness, kurtosis and discrimination indices of each item were calculated. Items with extreme asymmetry, kurtosis, or a discrimination index below 0.30 were removed from the scale. Regarding the discrimination indices, all the items discriminate satisfactorily with discrimination indices above 0.30 [32].

Four measurement models were compared: Model 1 (SF8), an eight-factor model using the original distribution of the questionnaire items; Model 2 (SF8b), corresponding to the factor structure of the previous model excluding the items with the lowest saturation in each factor; Model 3 (SF8c), an eight-factor model consistent with the results of the previous model, adding two second order factors as a solution to the poor discriminant validity of six of the factors; and Model 4 (SF4), an alternative solution to the previous model, responds to a four-factor structure, without second order factors, grouping the items of factors physical role, social function and emotional role in a single factor called social health. Items with low saturation in this new factor were also eliminated, as well as items of the factors general health, vitality and mental health in another factor called personal health. The estimation method used was Maximum Likelihood, following the recommendation of Thompson (2004) [33], in the sense that when confirmatory factor analysis is used, not only the fit of a theoretical model should be corroborated, but it is advisable to compare the fit indices of several alternative models to select the best one.

Confirmatory factor analyses were conducted using AMOS 21 software [34]. Variances in terms of error were specified as free parameters; for each latent variable (factor), a structural coefficient was set to one to make sure the scale was equal to one of the observable variables (items). Maximum credibility was chosen as the estimation method following Thompson [33], verifying the fit of a theoretical model, while comparing the fit indices of alternative models in order to select the model with the best fit.

To assess model fit, chi-square, the GFI (Goodness-of-Fit Index), and the RMSEA (Root Mean Square Error of Approximation) were selected as absolute fit measures. AGFI (Adjusted Goodness of Fit Index), the TLI (Tucker–Lewis Index), the CFI (Comparative Fit Index) were selected as measures of incremental fit. The χ^2^/DF (Chi-Squared fit Index divided by Degrees of Freedom) and the AIC (Akaike Information Criterion) were selected as parsimony measures [35,36].

Following Abalo et al. [28], an analysis of factorial invariance of the questionnaire was performed for the two subsamples, based on the best measurement model resulting from the prior stage. Cronbach’s alpha [37,38] and Omega coefficient [39,40] were used to calculate reliability for each dimension of the measurement models.

## 3. Results

### 3.1. Descriptive Analyses and Discrimination Indices

Results from the descriptive analyses and the discrimination indices (corrected total-item correlation) of each of the 36 items on the questionnaire for the total sample reflect mean scores that range between 4.55 and 8.71, and standard deviation values higher than 2.00 on all cases (within a response range between 0 and 10). All skewness and kurtosis values ranged from ±2.0 and ±3.5; therefore, normality is assumed for the variables. In addition, all items show discrimination indices above 0.45 (i.e., good discrimination) [32].

### 3.2. Confirmatory Factor Analyses

Overall, the results of the confirmatory factor analysis in the first subsample (GFI = 0.799; RMSEA = 0.073; CFI = 0.895) and the second subsample (GFI = 0.783; RMSEA = 0.078; CFI = 0.888) for the SF8 model correspond to an eight-factor structure according to the original distribution of the items, indicating that the measurement model, in both subsamples, is unacceptable (Table 1).

The eight factors of the SF8 model explain approximately 71% and 73% of the variance in the first and second subsamples, respectively. On the other hand, 8 of the 36 items show saturations below 0.70 in their predicted dimensions, (items 1, 3, 12, 23, 24, 26, 30 and 33) in the first subsample and 6 (items 1, 3, 12, 23, 24 and 26) in the second subsample. In both subsamples, high intercorrelations among the general health, vitality and mental health factors were observed, and among the factors physical role, social function and emotional role, not very adequate discriminant validity was evidenced among them.

The overall results from the confirmatory factor analyses for the first (GFI = 0.935, RMSEA = 0.042, CFI = 0.978) and second subsamples (0.917 GFI, RMSEA 0.054, CFI 0.967) of the second model that was assessed (SF8b), corresponding to the structure of the previous model without the items of lower saturation in each factor, indicate that this measurement model is better than the previous model, showing an acceptable fit (Table 1). The factors in this model together account for approximately 80% of the variance in the first and second subsamples. On the other hand, in both subsamples, all items except items 1 and 24 saturate above 0.70 in their predicted dimension. Again, high intercorrelations were observed between factors 2, 6 and 7, and between factors 4, 5 and 8, evidencing poor discriminant validity among the factors.

**Figure 1:**
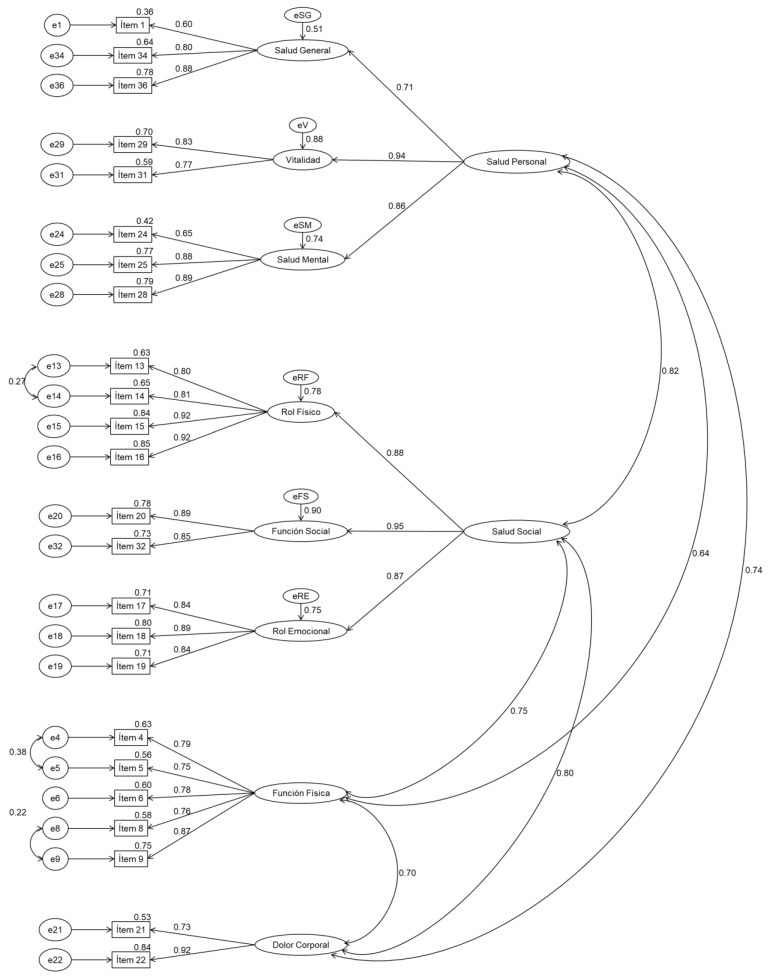
Measurement model SF8b for the SF36 questionnaire. Confirmatory factor analysis Subsample 1.

**Figure 2:**
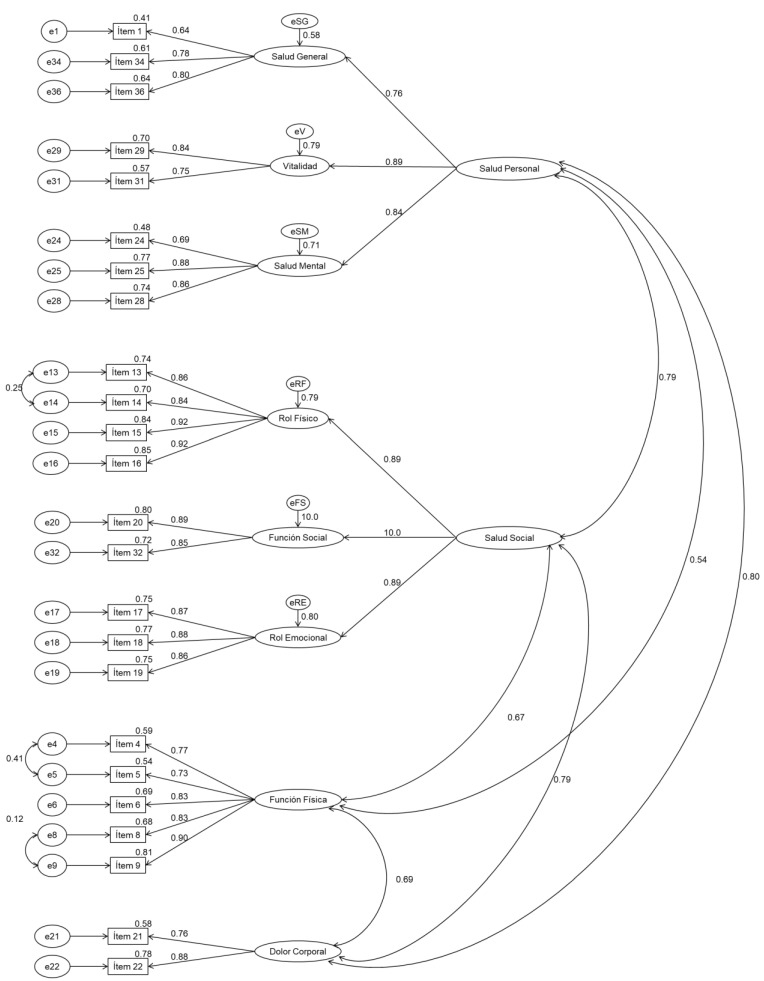
Measurement model SF8b for the SF36 questionnaire. Confirmatory factor analysis Subsample 2.

For the third model tested (SF8b), the overall results of confirmatory factor analyses on the first (0.920 GFI, RMSEA 0.054, CFI 0.969) and second subsamples (0.888 GFI, RMSEA 0.065, CFI 0.947), corresponding to the structure of the previous model adding two second order factors, indicate that this model of measurement is similar to the previous model (Table 1). Together, the factors in this model account for approximately 80% of the variance in both the first and second subsamples. On the other hand, according to Figure 1 and Figure 2, all items except items 1 and 24 saturate above 0.70 in their predicted dimension.

Results from the confirmatory factor analysis on the first (GFI = 0.966, RMSEA = 0.042, CFI = 0.987) and second subsample (GFI = 0.956, RMSEA = 0.058, CFI = 0.977) for the fourth and final model that was assessed (SF4), an alternative solution to the previous model that responds to a four-factor structure, without second order factors, indicate that this model of measurement is better than the previous model and that it has an optimal fit (Table 1). Together, the factors in this model account for more than 75% of the variance in the first and second subsamples. On the other hand, according to Table 2, except for item 24, all items show saturations above 0.70 in their predicted dimension (for both subsamples).

### 3.3. Invariance of the Factor Structure between Subsamples

The resulting fit indices (Table 3) allow for acceptance of the equivalence of the baseline measurement models between the first and second subsamples. Even though the Chi-square exceeds the required value to accept the invariance hypothesis, the GFI = 0.961, CFI = 0.982, RMSEA = 0.036 and AIC = 391.981 indices contradict this conclusion and allow us to accept the baseline model invariance (i.e., unrestricted model).

The metric invariance was characterized by adding restrictions to the baseline model on the factor loadings. The values shown in Table 3 allow for the acceptance of this level of invariance. Both the goodness of fit index (GFI = 0.960) and root mean square error of approximation (RMSEA = 0.034) show consistency for this direction.

The Akaike Information Criterion (AIC = 380.191) and Bentler comparative fit index (CFI = 0.982) do not vary greatly over those from the previous model. The criteria for assessment of nested models were proposed by Cheung and Rensvold [41], who suggest that if the difference of the CFI from both nested models diminishes in 0.01 or less, the restricted model is taken for granted and, thus, complies with the factorial invariance. The difference of the obtained CFIs allows acceptance of the metrical invariance model. We can conclude that factor loadings are equivalent in the first and second subsamples.

Having demonstrated the metric invariance between the subsamples, we assessed the equivalence between intercepts (strong factorial invariance). The indices (Table 3) show a good fit of this model, analyzed independently and nested within the metric invariance model. The difference between the two Bentler comparative fit indices was less than 0.001; the general fit index was 0.957; and the root mean square error of approximation was 0.033. Once the strong invariance was accepted, the two models were considered equivalent on both the factor coefficients and intercepts.

Most of the obtained factors from the confirmatory factor analysis showed internal consistency values above 0.70 for both samples, providing evidence of adequate internal consistency, especially when considering the small number of items (Table 4).

## 4. Discussion

The present research had two main goals: the first was to assess the psychometric properties of the SF-36 Health Questionnaire (construct validity and internal consistency) in the Mexican elderly population using confirmatory factor analysis CFA; the second goal was to calculate the factorial invariance of the questionnaire in two random, independent samples (i.e., cross-validation). The confirmatory factor analyses performed for each subsample support the four-factor structure—Physical Function, Body Pain, Physical Role and Psychological Health—as well as evidence an adequate internal consistency, especially when considering the reduced number of items in each of them; in addition, the factors, in general, show adequate saturations [37].

Results also provide strong evidence of cross-validation of the scale and, thus, of the stability of the structure.

However, the obtained model differs to some extent with that proposed by Alonso, Prieto and Antó [30], because in order to obtain a better fit and greater discrimination capacity, 23 of the 36 analyzed items were eliminated and the original saturation of some of the items changed, the latter being based on high correlations between factors, the modification indices of the confirmatory factor analyses and their theoretical justification.

However, it should be noted that the physical function and physical pain components are maintained, while the physical role, social function and emotional role components are summarized in physical role and the components general health, vitality and mental health in the psychological health component (named like this in this investigation for the content of the items that correspond to the components). In this way, we refer to the perceptual, cognitive, emotional and behavioral components described by various authors who have found evidence to support a multifactor model for this type of questionnaire.

Thus, more research is needed to corroborate or provide evidence contrary to the data that have been collected up to this point.

Promoting a better quality of life among older adults undoubtedly contributes to their health and psychological wellbeing, hence the need for reliable and valid instruments for its measurement. The present study therefore analyzes the psychometric properties of the SF-36 Health Questionnaire [30]. This study also serves as a premise for future research on measurement instruments for the perception of quality of life in populations with different personal and cultural factors. Finally, this instrument will be very useful for application in different areas such as, for example, descriptive or intervention studies.

The present study has four limitations, however. First, all the participants are older adults from an urban environment, which poses a threat to the generalizability of the findings. Extending the research to older adults in rural areas, as well as other regions and various age groups, is an area of opportunity for future studies.

Second, the instrument used is a self-report measure, which may be affected by social desirability bias. The third limitation is related to the fact that there were various simultaneous adaptations made to the original version of the questionnaire (i.e., translation, change in the response scale and applying it on a computer). As these changes were made at the same time, we were unable to detect whether any one of them could have affected the reliability and validity of the original questionnaire.

The fourth limitation has to do with the sample selection method, which, as it is not probabilistic, introduces the risk of a statistical bias in the results, so it is recommended to take the results with caution.

Likewise, it is necessary to assess whether the questionnaire predicts psychological wellbeing, satisfaction with life, physical condition, among others.

## 5. Conclusions

In summary, the analysis of the psychometric properties of the questionnaire shows that a four-factor structure is viable and adequate. The four-factor structure shows adequate fit, reliability and validity indices. However, future research should focus on confirming the obtained factor structure; this would provide stronger evidence with regard to the structure of the measure. Specifically, researchers should assess whether or not the invariance of the factor structure is met when considering, for example, gender, age, elderly of different cultures, among others.

## Figures and Tables

**Table 1:** Results from the generated models for both subsamples, absolute, incremental and parsimony fit indices.

	Absolute Indices			Incremental Indices			Parsimony Indices	
Model	χ^2^	GFI	RMSEA	AGFI	TLI	CFI	χ^2^/DF	AIC
SF8	1908.467 *	0.799	0.073	0.761	0.883	0.895	3.587	2104.467
SF8b	408.511 *	0.935	0.042	0.911	0.973	0.978	1.848	566.511
SF8c	500.535 *	0.920	0.048	0.899	0.964	0.969	2.112	626.535
SF4	102.286 *	0.966	0.042	0.945	0.982	0.987	1.860	174.286
SF8	2108.969 *	0.783	0.078	0.743	0.875	0.888	3.964	2304.969
SF8b	530.909 *	0.917	0.054	0.887	0.958	0.967	2.402	688.909
SF8c	724.576 *	0.888	0.065	0.859	0.939	0.947	3.057	850.576
SF4	145.695 *	0.956	0.058	0.927	0.968	0.977	2.649	217.695

Note: * *p* < 0.05; GFI = goodness of fit index; RMSEA = root mean square error of approximation; SRMR = Standardized Root Mean Square Residual; AGFI = adjusted goodness of fit index; TLI = Tucker–Lewis index; CFI = comparative fit index; CMIN/DF = chi-squared fit index divided by degrees of freedom; AIC = Akaike information criterion.

**Table 2:** Confirmatory factor analyses for the SF4. Model. Subsamples 1 and 2 (standardized solutions).

Item	Subsample 1				Subsample 2			
	F1	F2	F3	F4	F1	F2	F3	F4
4. How much does your current health status limit you to doing moderate activities, such as moving a table, pushing a vacuum cleaner, playing bowling or golf, working in the garden, or walking for more than an hour?	0.80				0.77			
5. How much does your current health condition limit you to lift or charge the market purchases?	0.75				0.73			
6. How much does your current health status limit you to climb several floors up the ladder?	0.78				0.83			
8. How much does your current health status limit you to bend, kneel, or bend?	0.76				0.83			
9. How much does your current health status limit you to walk a mile or more?	0.86				0.90			
21. During the last month, how often did you have pain in any part of the body?		0.73				0.77		
22. During the past month, how often has pain made your usual work difficult?		0.91				0.88		
13. During the last month, because of your physical health, did you reduce the amount of time you spend at work or other activities?			0.80				0.80	
15. During the last month, because of your physical health, have you stopped doing your daily activities?			0.83				0.85	
17. During the last month, because of an emotional problem, did you reduce your time spent at work or your daily activities?			0.70				0.75	
24. During the past month, how often have you been feeling nervous?				0.61				0.64
25. During the past month, how often have you felt so disheartened that nothing could encourage you?				0.76				0.75
29. During the last month, how often have you felt exhausted?				0.78				0.72
F1	-				-			
F2	0.70	-			0.69	-		
F3	0.77	0.77	-		0.73	0.75	-	
F4	0.60	0.71	0.75	-	0.49	0.80	0.74	-

**Table 3:** Goodness of fit indices for each of the assessed models.

Model	Fit Indices						
	χ2	gl	GFI	NFI	CFI	RMSEA	AIC
Model without restrictions	247.981 *	110	0.961	0.968	0.982	0.036	391.981
Metric Invariance	254.191 *	119	0.960	0.967	0.982	0.034	380.191
Strong factor invariance	268.097 *	129	0.957	0.966	0.982	0.033	374.097

Note: * *p* < 0.05; GFI = goodness-of-fit index; NFI = normed fit index; CFI = comparative fit index; RMSEA = root mean square error of approximation; AIC = Akaike information criterion.

**Table 4:** Omega and alpha coefficients for the factors obtained in the confirmatory factorial analyses, subsamples 1 and 2.

Factor	Subsample 1		Subsample 2	
	Ω	α	Ω	α
Physical Function	0.893	0.901	0.907	0.913
Body Pain	0.808	0.798	0.811	0.806
Physical Role	0.821	0.832	0.843	0.861
Psychological health	0.762	0.784	0.747	0.773

**Figure A1:**
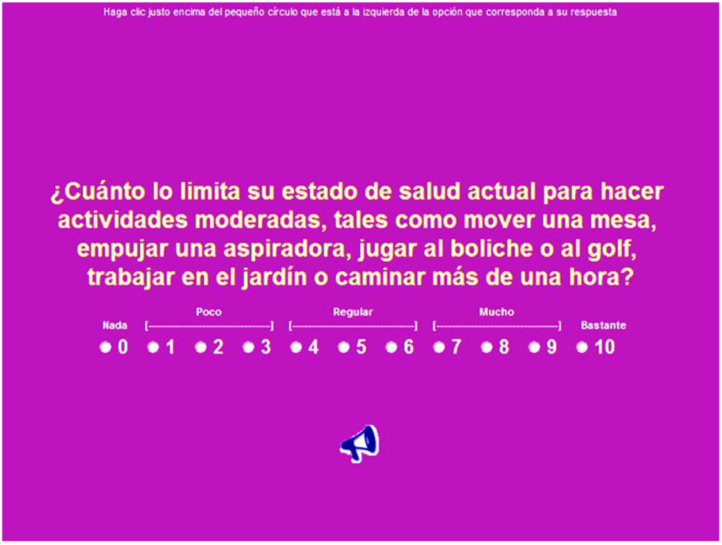
Example of an item response screen (It is part of a software that has Spanish as its native language).

## Data Availability

Data available upon request from correspondence author.

## References

[1] Long S., Sudnongbua S. (2017). Quality of life among elderly people in kampong cham province, Cambodia. Southeast Asian J. Trop. Med. Public Health.

[2] Chaves-García M., Sandoval-Cuellar C., Calero-Saa P. (2017). Asociación entre capacidad aeróbica y calidad de vida en adultos mayores de una ciudad colombiana. Rev. Peru. Med. Exp. Salud Publica.

[3] Molzahn A., Skevington S.M., Kalfoss M., Makaroff K.S. (2010). The importance of facets of quality of life to older adults: An international investigation. Qual. Life Res..

[4] Panday R., Kumar P. (2017). Quality of life among elderly living in old age home: A brief overview. Delhi Psychiatry J..

[5] Robles Y., Saavedra J., Mezzich J., Sanez Y., Padilla M., Mejía O. (2010). Índice de calidad de vida: Validación en una muestra peruana. An. Salud Ment..

[6] Sánchez R., García M., Martínez B. (2017). Encuesta de Salud SF-36: Validación en Tres Contextos Culturales de México. Rev. Iberoam. Diagnóstico Evaluación—Avaliação Psicológica RIDEP.

[7] Vagetti G., Barbosa V., Moreira N., De Oliveira V., Mazzardo O., De Campos W. (2014). Association between physical activity and quality of life in the elderly: A systematic review, 2000–2012. Rev. Bras. Psiquiatr..

[8] Wikman A., Wardle J., Steptoe A. (2011). Quality of Life and Affective Well-Being in Middle-Aged and Older People with Chronic Medical Illnesses: A Cross-Sectional Population Based Study. PLoS ONE.

[9] Baernholdt M., Hinton I., Yan G., Rose K., Mattos M. (2012). Factors associated with quality of life in older adults in the United States. Qual Life Res..

[10] Lins L., Carvalho F.M. (2016). SF-36 total score as a single measure of health-related quality of life: Scoping review. SAGE Open Med..

[11] Vilagut G., Ferrer M., Rajmil L., Rebollo P., Permanyer-Miralda G., Quintana J.M., Santed R., Valderas J.M., Ribera A., Domingo-Salvany A. (2005). El Cuestionario de Salud SF-36 español: Una década de experiencia y nuevos desarrollos. Gac. Sanit..

[12] Alonso J., Ferrer M., Gandek B., Ware J.E., Aaronson N.K., Mosconi P., Rasmussen N., Bullinger M., Fukuhara S., Kaasa S. (2004). Health-related quality of life associated with chronic conditions in eight countries: Results from the International Quality of Life Assessment (IQOLA) Project. Qual. Life Res..

[13] Bunevicius A. (2017). Reliability and validity of the SF-36 Health Survey Questionnaire in patients with brain tumors: A cross-sectional study. Health Qual. Life Outcomes.

[14] Doosti-Irani A., Nedjat S., Nedjat S., Cheraghi P., Cheraghi Z. (2019). Quality of life in Iranian elderly population using the SF-36 questionnaire: Systematic review and meta-analysis. East. Mediterr. Health J..

[15] Kang K., Gholizadeh L., Inglis S.C., Han H.-R. (2019). Validation of the Korean Version of the MacNew Heart Disease Health-Related Quality of Life Questionnaire. J. Nurs. Res..

[16] Salim S., Yamin M., Alwi I., Setiati S. (2017). Validity and Reliability of the Indonesian Version of SF-36 Quality of Life Questionnaire on Patients with Permanent Pacemakers. Acta Med. Indones.

[17] Farajzadeh M., Ghanei G., Sayehmiri K. (2017). Health Related Quality of Life in Iranian Elderly Citizens: A Systematic Review and Meta-Analysis. Int. J. Community Based Nurs. Midwifery.

[18] Garratt A.M., Stavem K. (2017). Measurement properties and normative data for the Norwegian SF-36: Results from a general population survey. Health Qual. Life Outcomes.

[19] Verdam M., Oort F.J., Sprangers M. (2016). Using structural equation modeling to detect response shifts and true change in discrete variables: An application to the items of the SF-36. Qual. Life Res..

[20] White M.K., Maher S.M., Rizio A.A., Bjorner J.B. (2018). A meta-analytic review of measurement equivalence study findings of the SF-36^®^ and SF-12^®^ Health Surveys across electronic modes compared to paper administration. Qual. Life Res..

[21] Cruz A., Guzmán C., Arriaga R.M., Colorado M., Morales F., Baeza G.d.C. (2019). Calidad de vida en adultos mayores con diabetes mellitus tipo 2 en un centro de salud en Villahermosa, Tabasco, México. Atención Fam..

[22] Peña-Marcial E., Bernal-Mendoza L.I., Reyna-Avila L., Pérez-Cabañas R., Onofre-Ocampo D., Cruz-Arteaga I., Silvestre-Bedolla D. (2019). Calidad de vida en adultos mayores de Guerrero, México. Univ. Salud.

[23] Salazar-Estrada J.G., López-Espinoza A., Ramírez-Ramírez S. (2018). Índice de masa corporal y calidad de vida en médicos de atención primaria en guadalajara, Jalisco, México. Actual. Nutr..

[24] Salazar-Estrada J.G., Martínez Moreno A.G., Torres López T.M., Aranda C., López-Espinoza A. (2016). Calidad de vida relacionada con la salud y obesidad en trabajadores de manufacturas en Jalisco, México. Arch. Latinoam. Nutr..

[25] Durán-Arenas L., Gallegos-Carrillo K., Salinas-Escudero G., Martínez-Salgado H. (2004). Hacia una base normativa mexicana en la medición de calidad de vida relacionada con la salud, mediante el Formato Corto 36. Salud Pública México.

[26] Zúniga M., Carrillo-Jiménez G., Fos P., Gandek B., Medina-Moreno M. (1999). Evaluation of health status using Survey SF-36: Preliminary results in Mexico. Salud Publica Mex..

[27] Montero I., León O. (2005). Sistema de clasificación del método en los informes de investigación en Psicología. Int. J. Clin. Health Psychol..

[28] Abalo J., Lévy J., Rial A., Varela J., Lévy J. (2006). Invarianza factorial con muestras múltiples. Modelización con Estructuras de Covarianzas en Ciencias Sociales.

[29] Schumacker R.E., Lomax R.G. (2010). A Beginner’s Guide to Structural Equation Modeling.

[30] Alonso J., Prieto L., Antó J. (1995). La versión española del SF-36 Health Survey (Cuestionario de Salud SF-36): Un instrumento para la medida de los resultados clínicos. Med. Clínica.

[31] Blanco H., Ornelas M., Tristán J.L., Cocca A., Mayorga-Vega D., López-Walle J., Viciana J. (2013). Editor for creating and applying computerise surveys. Procedia Soc. Behav. Sci..

[32] Brzoska P., Razum O. (2010). Validity Issues in Quantitative Migrant Health Research: The Example of Illness Perceptions.

[33] Thompson B. (2004). Exploratory and Confirmatory Factor Analysis. Understanding Concepts and Applications.

[34] Arbuckle J.R. (2012). AMOS Users Guide Version 21.0.

[35] Byrne B.M. (2010). Structural Equation Modeling with AMOS: Basic Concepts, Applications, and Programming.

[36] Gelabert E., García-Esteve L., Martín-Santos R., Gutiérrez F., Torres A., Subirà S. (2011). Psychometric properties of the Spanish version of the Frost Multidimensional Perfectionism Scale in women. Psicothema.

[37] Elosua P., Zumbo B.D. (2008). Coeficientes de fiabilidad para escalas de respuesta categórica ordenadas. Psicothema.

[38] Nunnally J.C., Bernstein I.H. (1995). Teoría Psicométrica.

[39] Revelle W., Zinbarg R.E. (2009). Coefficients alpha, beta, omega and the glb: Comments on Sijtsma. Psychometrika.

[40] Sijtsma K. (2009). On the use, the misuse, and the very limited usefulness of Cronbach’s alpha. Psychometrika.

[41] Cheung G.W., Rensvold R.B. (2002). Evaluating goodness-of-fit indexes for testing measurement invariance. Struct. Equ. Modeling.

